# Optic Nerve Head Hemoglobin Levels in Glaucoma: A Structural and Functional Correlation Study

**DOI:** 10.1155/2021/9916102

**Published:** 2021-10-07

**Authors:** Janaina A. G. Rocha, Diego T. Dias, Maria Betânia C. Lemos, Fábio N. Kanadani, Augusto Paranhos, Carolina P. B. Gracitelli, Tiago S. Prata

**Affiliations:** ^1^Department of Ophthalmology, Federal University of São Paulo, São Paulo, Brazil; ^2^Glaucoma Unit, Opty Group Brazil, São Paulo, Brazil; ^3^Instituto de Olhos Ciências Médicas—IOCM, Belo Horizonte, Brazil; ^4^Hospital de Olhos de Sergipe—HOS, Aracaju, Brazil; ^5^Department of Ophthalmology, Mayo Clinic, Jacksonville, FL, USA; ^6^Centro de Estudos Alcides Hirai, Ver Mais Oftalmologia, Vinhedo, São Paulo, Brazil

## Abstract

**Purpose:**

To investigate structural and functional correlations in glaucoma patients using optic nerve head hemoglobin (ONH Hb) measurements as determined by automated colorimetric analysis of conventional retinography.

**Methods:**

We prospectively enrolled healthy participants and glaucomatous patients with a wide range of disease stages. All participants underwent visual field (VF) testing (standard automated perimetry, SAP), color fundus imaging (mydriatic retinography), and peripapillary retinal nerve fiber layer (pRNFL) assessment through spectral-domain optical coherence tomography (SD-OCT). Software Laguna ONhE was used to estimate the amount of ONH Hb and to determine the glaucoma discriminant function (GDF) index. Scatter plots were constructed, and regression analysis was used to investigate the correlations between GDF, average pRNFL thickness, and VF mean deviation (VFMD) index values. A secondary analysis was performed to compare each parameter between three different glaucoma groups divided according to VFMD values (mild, >−6 dB; moderate, −6 to −12 dB; and advanced, <−12 dB).

**Results:**

One hundred ninety-six eyes from 123 participants (69 with glaucoma and 54 controls) were enrolled. Overall, all parameters evaluated differed significantly between glaucomatous and control eyes (*p* ≤ 0.001). The comparison of each parameter according to groups of disease stages revealed significant differences between controls and each of the glaucomatous groups (*p* < 0.001). More pronounced changes in GDF values were observed in early disease stages. We found significant nonlinear correlations between GDF and VFMD values (*R*^2^ = 0.295, *p* < 0.001) and between pRNFL thickness and VFMD (*R*^2^ = 0.598, *p* < 0.001). A linear correlation was found between GDF and pRNFL thickness values (*R*^2^ = 0.195, *p* < 0.001).

**Conclusion:**

Our results showed significant associations between ONH Hb values and both structural and functional damage in glaucoma obtained by SD-OCT and SAP, respectively. The nonlinear correlation we found and the GDF behavior along different disease stages suggest that ONH Hb levels' reduction may precede visual function changes in early glaucoma stages.

## 1. Introduction

Glaucoma consists in the main cause of irreversible blindness worldwide [1]. The disease is considered as a progressive and chronic optic neuropathy, characterized by specific changes on the optic nerve head (ONH), peripapillary retinal nerve fiber layer (pRNFL), and visual field (VF) [2, 3]. Disease control and blindness prevention are strictly related to early diagnosis [2, 3]. However, the diagnosis of glaucoma can be challenging in the early stages of the disease, especially for general ophthalmologists [4, 5]. Taking this into account, it is essential to perform the correlation between structural and functional changes. Anatomical evaluation can be performed through stereoscopic retinography [6] and automated quantitative exams such as optical coherence tomography (OCT) which were developed with the aim of contributing to the diagnosis of the disease [7–9]. Nevertheless, the high cost can represent a limitation to the access of the referred diagnostic tools.

Considering the pathological mechanisms of glaucoma, vascular dysfunction has been related to the optic nerve glaucomatous lesion [10]. The access to these vascular changes can be achieved through some diagnostic tests. At first, one can mention the evaluation of the ocular blood flow, through nearby vessels, using echo Doppler [11]. Other tests were developed to measure oxygen concentration in the optic nerve [12], blood flow, and vascular structure with the emergence and clinical application of OCT angiography (OCT-A) [13].

Previous studies have evaluated the hypothesis of a relationship between tissue perfusion and the level of hemoglobin (Hb) and oxygenation. Tissues with adequate perfusion demonstrate a good level of Hb, whereas low levels occur in tissue loss [14, 15]. Some studies have proposed a simple method for measuring hemoglobin levels in the ONH, assessing conventional retinography through automated colorimetric analysis, using software Laguna ONhE [16–19]. These preceding data have demonstrated that lower levels of optic nerve head hemoglobin (ONH Hb) are found in patients with established glaucoma, along with high reproducibility results, both in glaucomatous and nonglaucomatous eyes [15].

All these considered, we sought to investigate the correlation between the levels of ONH Hb, assessed by automated colorimetric analysis, and the levels of structural and functional damage, obtained by spectral-domain optical coherence tomography (SD-OCT) and standard automated perimetry (SAP), respectively, in glaucomatous patients.

## 2. Methods

This study protocol, according to the tenets of the Declaration of Helsinki, was approved by the ethics committee and the institutional review board of the Federal University of São Paulo (CEP: 4.055.180). Written informed consent was obtained by all participants prior to enrollment and examination.

### 2.1. Participants

In this observational cross-sectional study, we included consecutive healthy individuals and patients with primary open-angle glaucoma attending to the Glaucoma Sector of Hospital Medicina dos Olhos (São Paulo, Brazil) between May 2020 and January 2021.

Glaucoma was defined as the presence of glaucomatous optic neuropathy (GON) associated or not with the corresponding VF alteration. The criteria used to define the disease were the same as those used by our research group in previous studies [20, 21]. GON was considered in the presence of a vertical cup-to-disc ratio (VCDR) greater than or equal to 0.6, asymmetry of VCDR between the eyes (greater than 0.2), detection of localized or diffuse pRNFL defects, or neuroretinal rim defects, without other pathologies that could explain these changes. We adopted VF glaucomatous defect in the SAP (Humphrey SITA—Standard 24-2, Carl Zeiss Meditec, Dublin, CA), if there were, on the pattern deviation plot, three or more points in clusters with a probability of less than 5% (points directly above and below the blind spot or on the edge of the field were excluded), a pattern standard deviation index with a probability of less than 5%, or the result outside the normal limits on glaucoma hemifield test.

The following exclusion criteria were adopted: age ≤18 years, previous ocular trauma or posterior segment intraocular surgery, significant media opacity, difficulty in performing the exams, diagnosis of primary angle closure or secondary glaucoma, and presence of ocular diseases other than glaucoma that could influence the results, such as diabetic or hypertensive retinopathy and macular edema.

Regarding the control group, nonglaucomatous patients were included, demonstrating normal appearance of the optic disc, such as a VCDR less than 0.6, absence of defects on the neuroretinal rim or pRNFL, and intraocular pressure (IOP) less than 21 mmHg, without treatment [20].

### 2.2. Study Protocol

Complete ophthalmological examination was performed in all participants. This evaluation included clinical history, best-corrected visual acuity, slit-lamp biomicroscopy, IOP measurement with Goldmann applanation tonometry, gonioscopy, ultrasound pachymetry, dilated fundus examination, VF testing (Humphrey SITA—Standard 24-2, Carl Zeiss Meditec, Dublin, CA), color fundus imaging (mydriatic fundus retinography Canon CR-2; Canon, Tokyo, Japan), and pRNFL and topographic ONH measurements based on SD-OCT (RTVue-100 OCT; Optovue Inc., Fremont, CA).

Accepted reliability indices for this protocol include patients' experience in performing VF testing (at least 3 previous exams). Patients were excluded from the study if the exams presented >15% false positives or >33% loss of fixation or false negative. Additionally, during SAP review, the exam was eliminated in the presence of some artifacts such as edge defects, inattention or loss of fixation, fatigue effect, or alterations indicative of pathologies other than glaucoma.

**Figure 1:**
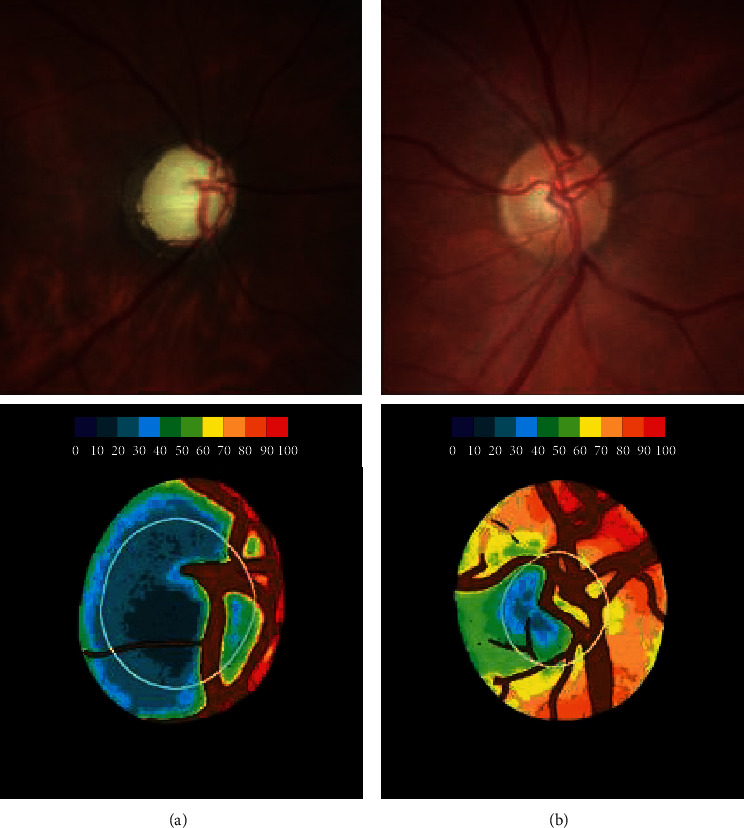
Examples of the optic nerve head: glaucomatous (a) and normal (b). Retinographies of the optic disc are represented in the upper images, and their pseudo-images referring the amount of hemoglobin are present in the lower images. A colorimetric scale (at the top of the lower images) indicates the amount of hemoglobin correspondent.

The color fundus retinography was then analyzed by Laguna ONhE software. The full description of the program was presented in a previous study [16]. Summarizing, Laguna ONhE analyzes conventional fundus photographs to measure the amount of ONH Hb. Software considers three spectral components of ONH photographs: red, green, and blue. The red component is reflected by ONH areas with a high Hb content. On the contrary, a smaller proportion of the red light, compared to green and blue components, is reflected in areas with low Hb content. The analysis of various formulas, based on the reflected amounts of red, green, and blue light, was almost linearly proportional to the amount of Hb present [16].  Figure 1 demonstrates examples of patients with normal and glaucomatous papilla and the respective pseudo-images indicating the Hb levels. Finally, software determines an index of glaucoma discriminant function (GDF), based on colorimetric analysis and OHN Hb levels [16]. This index was performed by dividing the ONH into 3 concentric rings, and each one of them was divided into 8 parts, a total of 24 sectors. The sectors that showed the greatest difference in the amount of Hb, between eyes of patients with glaucoma and control, were those located in the vertical region, especially sectors 8 and 20, as shown in a previous study. From this, the GDF takes into account the slope of the Hb amount with the mean of these specific sectors (8 and 20) presenting 89% sensitivity at 95% specificity [16].

### 2.3. Sampling and Statistical Analysis

The sample size was calculated to estimate the correlation between GDF and pRNFL/VF mean deviation (VFMD). At the significance level of 5% and minimum power of 90% and considering a minimum value of 0.5 for correlation, 39 individuals are required in the sample.

Clinical and demographic data were demonstrated through descriptive analysis. The Shapiro–Wilk test was used to assess whether the data had a normal distribution. Normally distributed data were presented as mean and standard deviation, and nonnormally distributed data were presented as median and interquartile intervals. Regarding the comparison between groups, for continuous normally distributed variables, the independent samples *t*-test was performed, while for those nonnormally distributed, the Mann–Whitney test was used. The *χ*^2^ test was performed to compared categorical data. For structure-functional relationship evaluation, data of the glaucoma patients were analyzed, scatter plots were constructed, and regression analysis was used to investigate the correlations between GDF, SD-OCT average pRNFL thickness, and VFMD index values. Additionally, a correlation subanalysis considering disease stage was performed. Taking into consideration VFMD index values, patients were divided into 3 groups, mild (>−6 dB), moderate (−6 to −12 dB), and advanced glaucoma (<−12 dB), according to Hoddap et al. [22]. Computerized analysis was performed using R version 4.0.2. *p* value <0.05 was considered significant.

## 3. Results

A total of 196 eyes from 123 participants (69 patients with glaucoma and 54 controls) were included in this study. Twenty-four eyes from 16 patients were excluded from the analysis due to low reliability in the VF test or poor-quality images on retinography. There was no significant difference in age, gender, race, and IOP between the two groups (*p* ≥ 0.10  for all comparisons). Glaucoma patients present thinner central corneal thickness (CCT) compared to the control group (*p*=0.004). Visual field index (VFI), VFMD, and average pRNFL thickness differed significantly between patients and controls (*p* < 0.001 for all comparisons), as expected.  Table 1 provides clinical and ocular characteristics of included patients.

Analyzing the history of systemic comorbidities, especially the presence of cardiovascular risk factors (arterial hypertension and/or diabetes mellitus), patients with glaucoma and control did not show differences (*p*=0.32). There was no statistical difference related to GDF values in the presence or absence of these comorbidities, both in control patients (*p*=0.85) and in those with glaucoma (*p*=0.33). In concern to the use of topical hypotensive medications and GDF index values, we did not find any statistical difference between patients using beta-blockers and those who did not use them (*p*=0.10), as well as for the use of prostaglandin analogues (*p*=0.38) or alpha-adrenergic agonists (*p*=0.37).

**Figure 2:**
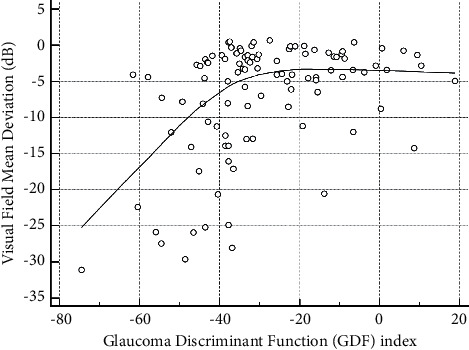
Significant nonlinear correlation between the glaucoma discriminant function (GDF) index and visual field mean deviation index values (*R*^2^ = 0.295; *p* < 0.001). A LOESS (local regression smoothing) trendline is plotted with a degree of smoothing of 80%.

**Figure 3:**
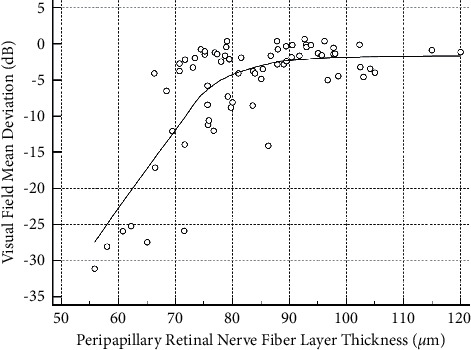
Significant nonlinear correlation between peripapillary retinal nerve fiber layer thickness and visual field mean deviation index values (*R*^2^ = 0.598; *p* < 0.001). A LOESS (local regression smoothing) trendline is plotted with a degree of smoothing of 80%.

**Figure 4:**
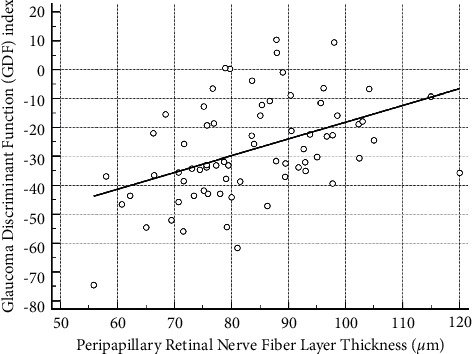
Significant linear correlation between peripapillary retinal nerve fiber layer thickness and glaucoma discriminant function (GDF) index values (*R*^2^ = 0.195; *p*=0.001).

Regarding the structure-function correlations we investigated in the glaucoma group, we found significant nonlinear correlations between GDF and VFMD values (*R*^2^ = 0.295, *p* < 0.001; Figure 2) and between OCT's pRNFL thickness and VFMD values (*R*^2^ = 0.598, *p* < 0.001; Figure 3). Additionally, a linear correlation was found between GDF and OCT's RNFL thickness values (*R*^2^ = 0.195, *p* < 0.001), as demonstrated in Figure 4.

The comparison of ONH Hb, pRNFL, and functional measurements between controls and glaucomatous eyes (divided according to disease stage; Table 2) revealed significant differences between controls and each of the glaucomatous groups (*p* < 0.001). In addition, although there was a significant difference regarding pRNFL thickness between eyes with moderate and advanced glaucoma, GDF values did not differ significantly between these two groups.

## 4. Discussion

Improved understanding of the structure-function relationship in patients with glaucoma is essential for diagnosis and monitoring of the disease. Within this scenario, in the last decades, the evolution of OCT imaging devices plays an important role in objective structural assessment. This approach provided significant information, especially regarding the description of both ONH and pRNFL parameters. Nonetheless, in some clinical situations related to intrinsic ocular characteristics or even technical difficulties (for example, high myopia, tilted discs, advanced glaucoma, or peripapillary atrophies), the use of conventional pRNFL analysis is limited [23–25]. Additionally, cost issues and portability often restrict access to this technology. In our study, we evaluated the relationship between the levels of structural and functional damage in glaucomatous patients, obtained by SD-OCT and SAP, respectively, and ONH Hb values, assessed in a low-cost, noninvasive manner by automated colorimetric analysis. Our results showed a significant association between the evaluated parameters.

There are scant data in the literature regarding structure-functional correlations using ONH Hb values. A previous study has demonstrated a significant correlation between anatomical damage (RNFL thickness) and hemoglobin content in specific sectors of the ONH [19]. In addition, considering functional findings, Gonzalez de la Rosa et al. showed a significant agreement between the GDF index and OCT parameters and perimetry results (Easyfield perimeter) [16]. Mendez-Hernandez et al. also found that the GDF index correlated well with Octopus perimetry indices and Spectralis OCT metrics [26]. It must be highlighted that Gonzalez-Hernandez and Saavedra not only reaffirmed the significant linear correlation between GDF and OCT parameters but also demonstrated the relationship between the indices obtained by Laguna ONhE to be curvilinear when compared to retinal function measured by VF testing [27]. We believe that our findings not only corroborate these initial structure-functional correlation results reported by Gonzalez-Hernandez and Saavedra [27] but also add significant information about different behaviors of each parameter along the disease spectrum. One may perceive (Table 2) that while mean pRNFL thickness gradually diminishes as the disease advances, most changes on GDF values were observed in the early stages of the disease (between controls and mild glaucoma). Conversely, functional status as determined by the VFMD tends to decay in the latter stages (moderate and advanced glaucoma) of the disease. Although this assumption needs further confirmation, it suggests that GDF performance would be more suitable in early disease stages.

We believe it is important to discuss the nature of our main findings. More specifically, what would be the reasons for the nonlinear relationship between ONH Hb measurements and functional status? Initially, the intrinsic evolution of structural-functional glaucomatous damage could partially explain this relationship. It is well known that axonal loss (structural changes) precedes VF loss (functional changes). As a result, especially in the early stages of the disease, structural deterioration may occur without VF correspondence [28–31]. Since the GDF index is an estimation of a more structural parameter, it could lead to the nonlinear structure-functional correlations as we found. It should also be considered that the VFMD index, which is derived from retinal sensitivity measurements, is based on a logarithmic scale, which also contributes to this curvilinear pattern.

At this point, it is important to discuss the main clinical implications of our findings. The knowledge of the relationship of glaucoma with some vasospasm phenomena [32, 33], such as migraines [34, 35], syndrome of Raynaud, and peripheral vascular dysregulation [36], and also with sleep apnea [37, 38], supports the idea of the participation of vascular dysfunction in the pathogenesis of glaucoma. However, despite the recent evolution in studies that assess blood flow in the ONH and especially vascular density through OCT-A, the high cost of this technology represents a limitation of its widespread use [12, 39–44]. Within this context, as previously described, the Laguna ONhE program represents a feasible lower cost option, analyzing conventional color retinography, to evaluate the vascular component by estimating Hb levels at the ONH. Although our study does not provide a direct comparison with other methods focusing specifically on vascular changes in the ONH, a recent study comparing the Laguna ONhE program and OCT-A demonstrated that the technologies showed similar performance in diagnosing patients with open-angle glaucoma. The area under the receiver operating characteristic curve for discriminating between glaucomatous and healthy eyes was 0.93 (95% CI: 0.86 to 0.97) for a specific OCT-A parameter and 0.92 (95% CI: 0.86 to 0.97) for the GDF index [45]. We believe the results of our study along with the existing literature support ONH Hb measurement as a viable and accessible tool for assessing structural damage in glaucoma, likely more related to the vascular component.

Our study presents some limitations and characteristics that should be mentioned. First, our findings should only be extrapolated to this specific population and therefore should not be applied to glaucomatous patients with different patterns. Second, we performed a cross-sectional study. Therefore, we were not able to evaluate the prognostic value of the GFD index nor investigate cause-effect relationships. Third, although it has been shown that age does not seem to significantly influence ONH Hb levels [17], it certainly impacts pRNFL thickness throughout life. This fact may have influenced, in part, our correlation analyses. Finally, some study patients had both eyes included in the analysis without any specific statistical adjustment. Even though this should be considered while interpreting our findings, we believe that such an adjustment would be more indicated for surgical studies, longitudinal analyses, or risk factor studies, rather than a cross-sectional structure-functional analysis as we present herein.

In conclusion, our results showed significant associations between ONH Hb values and both structural and functional damage in glaucoma obtained by SD-OCT and SAP, respectively. The nonlinear structure-functional findings and the GDF behavior along different disease stages suggest that ONH Hb levels' reduction may precede visual function changes in early glaucoma stages. Further longitudinal studies are warranted to evaluate the diagnostic performance of this technique in different types of glaucoma and as a tool for longitudinal monitoring of these patients.

## Figures and Tables

**Table 1:** Demographic and ocular characteristics of study patients.

Variables	Control group	Glaucoma group	*p* value
Age ± SD (years)	60.60 ± 14.21	63.54 ± 10.46	0.100
Gender (%, W/M)	68.5/31.5	59.4/40.6	0.300
Race (%, C/AD/O)	74.0/3.8/22.2	70.3/9.4/20.3	0.470
Intraocular pressure (mmHg)	13.00 (12.25, 15.00)	13.00 (12.00, 15.00)	0.950
Central corneal thickness (*μ*m)	537 (517, 553)	506 (482, 528)	0.004
pRNFL thickness (*μ*m)	103.74 ± 8.67	83.47 ± 13.06	<0.001
VFMD index (dB)	−0.76 ± 2.05	−7.23 ± 8.02	<0.001
VFI (%)	99 (98, 99)	95 (79.5, 97.5)	0.001
Spherical equivalent (D)	0.5 (−0.5, 2.0)	0.0 (−1.12, 1.37)	0.033
GDF index	16.25 ± 14.17	−28.02 ± 19.08	0.0001
VCDR	0.40 ± 0.18	0.75 ± 0.13	0.0001

Data are given as mean ± standard deviation whenever indicated. W: women; M: men; C: Caucasian; AD: African descendants; O: others; pRNFL: peripapillary retinal nerve fiber layer; VFMD: visual field mean deviation; VFI: visual field index; GDF: glaucoma discriminant function; VCDR: vertical cup-to-disc ratio.

**Table 2:** Glaucoma discriminant function index and average peripapillary retinal nerve fiber layer thickness by disease stage.

Variables^a^	Control group (*n* = 90)	Mild glaucoma (*n* = 66)	Moderate glaucoma (*n* = 13)	Advanced glaucoma (*n* = 25)	*p* value
GDF index	16.25 ± 14.17	−24.59 ± 16.50	−31.65 ± 15.40	−34.26 ± 24.90	<0.001^b^
pRNFL thickness (*μ*m)	102.27 ± 14.40	87.79 ± 11.68	77.24 ± 4.50	67.60 ± 8.84	<0.001^c^
VFMD index (dB)	−0.76 ± 2.05	−2.21 ± 1.67	−8.43 ± 1.67	−19.62 ± 6.17	<0.001^c^

^a^Data are given as mean ± SD. ^b^Each glaucoma group differed significantly from controls. There was also a significant difference between eyes with mild and advanced glaucoma. ^c^Each glaucoma group differed significantly from controls. There were also significant differences between eyes with mild and advanced glaucoma and between eyes with moderate and advanced glaucoma.

## Data Availability

The data used to support the findings of this study are included within the supplementary information file (Excel sheet).

## Supplementary Materials

Supplementary Materials ONH Hb and VFMD DATA_JO experimental data of the manuscript are included in the supplementary file.
